# Novel insights into the effect of vitamin B_12_ and omega-3 fatty acids on brain function

**DOI:** 10.1186/s12929-016-0241-8

**Published:** 2016-01-25

**Authors:** Richa Rathod, Anvita Kale, Sadhana Joshi

**Affiliations:** Department of Nutritional Medicine, Interactive Research School for Health Affairs, Bharati Vidyapeeth Deemed University, Pune Satara Road, Pune, 411043 India

**Keywords:** BDNF, Epigenetics, Homocysteine, Omega-3 fatty acids, One-carbon cycle, Vitamin B_12_

## Abstract

The prevalence of psychiatric disorders which are characterized by cognitive decline is increasing at an alarming rate and account for a significant proportion of the global disease burden. Evidences from human and animal studies indicate that neurocognitive development is influenced by various environmental factors including nutrition. It has been established that nutrition affects the brain throughout life. However, the mechanisms through which nutrition modulates mental health are still not well understood. It has been suggested that the deficiencies of both vitamin B_12_ and omega-3 fatty acids can have adverse effects on cognition and synaptic plasticity. Studies indicate a need for supplementation of vitamin B_12_ and omega-3 fatty acids to reduce the risk of cognitive decline, although the results of intervention trials using these nutrients in isolation are inconclusive. In the present article, we provide an overview of vitamin B_12_ and omega-3 fatty acids, the possible mechanisms and the evidences through which vitamin B_12_ and omega-3 fatty acids modulate mental health and cognition. Understanding the role of vitamin B_12_ and omega-3 fatty acids on brain functioning may provide important clues to prevent early cognitive deficits and later neurobehavioral disorders.

## Background

The escalating prevalence of brain disorders is currently a global health challenge [1] and has emerged as leading contributors to global disease burden [2]. Brain disorders affect neurological and cognitive performance and therefore have lifelong devastating effects on the individual, family and society. However, the underlying causes of mental health problems are poorly understood. Substantial evidence suggests that cognitive impairment can be influenced by number of environmental factors such as nutrition [3]. Nutrition plays a key role in maintaining optimal brain health throughout the lifespan of an individual [4]. In view of this, the studies examining the link between nutrition and mental health have gained widespread attention in recent years.

Nutrients like B vitamins and omega-3 fatty acids have been widely studied in recent years in context of brain development and functioning. In developing countries like India, due to widespread vegetarianism, vitamin B_12_ deficiency [5] coexists with suboptimal levels of omega-3 fatty acids [6]. Reports suggested that these nutrients are critical for brain health and their deficiencies could influence cognitive performance adversely. It has also been suggested that vegetarians should increase their dietary intake of vitamin B_12_ and omega-3 fatty acids to reduce increased risk factors for non-communicable diseases [7]. However, the effects of both vitamin B_12_ and omega-3 fatty acids supplementation together on neurological disorders are relatively unexplored and the underlying mechanisms need to be established.

In this review, we highlight the role of vitamin B_12_ and omega-3 fatty acids on brain function and proposed mechanisms through which these nutrients influence mental health and cognition.

## Review

### Vitamin B_12_

Vitamin B_12_ is generally found only in foods of animal origin [8]. Thus, the population predominantly consuming a vegetarian diet is deficient in vitamin B_12_ [5, 9]. Vitamin B_12_ is a key micronutrient required for proper brain development and is associated with one carbon metabolism that plays a pivotal role in transmethylation reactions. It is involved in the formation of S-adenosylmethionine (SAM), which is an important substrate for epigenetic mechanisms [10]. Vitamin B_12_ is known to have fundamental roles in the brain function at all ages and also in the prevention of disorders of CNS development, mood disorders and dementias including Alzheimer’s disease and vascular dementia in elderly people [11].

Elevated methylmalonic acid and total homocysteine concentrations are considered as sensitive metabolic markers for vitamin B12 deficiency [12]. Myelopathy and neuropathy are known to be the main clinical manifestations of vitamin B12 deficiency [13]. Symptoms of vitamin B12 deficiency include megaloblastic anaemia, tingling and numbness of the extremities, gait abnormalities, visual disturbances, memory loss and dementia [10]. Reports indicate that low dietary intake of vitamin B_12_, especially in pregnant women and in the elderly population are associated with developmental and neurological disorders [14, 15].

Studies indicate a need for supplementation of vitamin B_12_ to improve pregnancy outcome and reduce the risk of neurodevelopmental disorders [9]. Reports indicate a positive association between maternal vitamin B_12_ status and cognition in the offspring [16]. In contrast, a study in Indian school children at 6–10 years of age found an inverse association of maternal vitamin B_12_ concentrations with cognitive performance [17]. Our recent animal study has shown that vitamin B_12_ supplementation (50 μg/kg of diet) was able to maintain the levels of docosahexaenoic acid (DHA) and brain derived neurotrophic factor (BDNF) in the hippocampus and cortex, and sustain cognition in the adult rat offspring as compared to control animals (receiving 25 μg/kg of vitamin B_12_ in diet) [18]. A review by van de Rest et al. concludes that there are limited studies examining the association of maternal vitamin B_12_ with cognition and results are inconsistent suggesting a need for more research in this area [19].

### Omega-3 fatty acids

The role of omega-3 fatty acids especially DHA in brain development is gaining widespread attention [20]. The dietary sources of omega-3 fatty acids are fish and sea foods only [21] which are the rich sources of DHA. Hence, the vegetarian population particularly Asian Indians are found to be deficient in omega-3 fatty acids [22]. Further, over the past 150 years, the western diet has altered such that the ratio of omega-3 to omega-6 fatty acids has changed from 1:1 to 1:20–25 indicating that this diet is deficient in omega-3 fatty acids and is rich in omega-6 fatty acids [23]. Thus, the deficiency of omega-3 fatty acids and consumption of western diet has been suggested to be associated with cognitive impairment [24, 25].

There is increasing evidence which indicates the importance of omega-3 fatty acids in brain health across the lifespan [26]. DHA, which is the core member of omega-3 fatty acids, is highly concentrated in the brain and the outer segments of retinal rods and cones, constituting around 50 % of the total polyunsaturated fatty acids [27]. DHA participates in a number of neuronal processes including neurogenesis, neuroplasticity, neuron differentiation and survival, membrane integrity and fluidity [28]. A large body of evidence in animals has shown that maternal supplementation of DHA during gestation has neuroprotective effects against prenatal stress-induced brain dysfunction [29], hyperoxic injury [30] and hypoxic ischemic injury [31].

A recent study has reported an inverse association between intake of omega-3 fatty acids and depression [32]. DHA is well-implicated in synaptic transmission, synaptogenesis, learning and memory processes [33]. The density of dendritic spine has shown to be increased in the hippocampus of the animals with the oral supplementation of DHA [34]. Several evidences from animal and human studies have shown a positive association between DHA and cognitive development [35–38]. Thus, the role of omega-3 fatty acids in influencing brain health and wellbeing is well established however further investigation is required to better understand the underlying mechanisms and also to develop therapeutic targets for neurological disorders.

### Possible mechanisms of the effects of vitamin B_12_ and omega-3 fatty acids on brain development

**Fig. 1:**
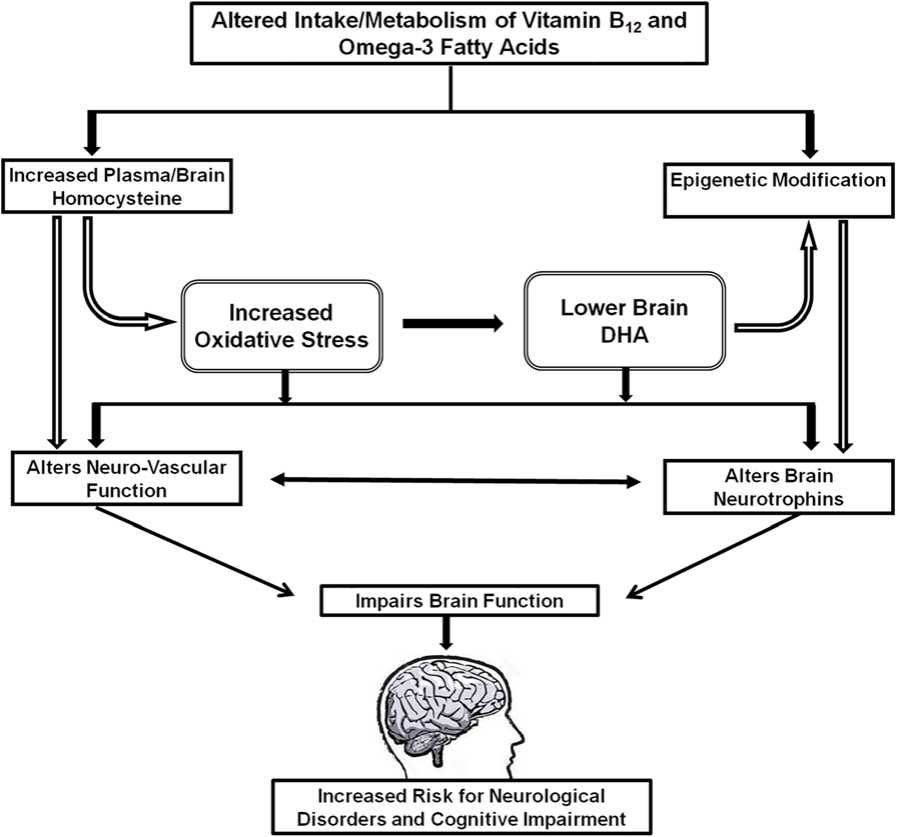
Proposed mechanisms through which vitamin B_12_ and omega-3 fatty acids affects brain functioning. Alerted intake/metabolism of vitamin B_12_ and omega-3 fatty acids affects brain function through multiple pathways. Increased homocysteine levels and altered epigenetic modification influences brain neurotrophins and neuro-vascular function directly or through increased oxidative stress and lower brain omega-3 fatty acid levels which may increase the risk for neurological disorders and cognitive impairment

The combined deficiency of both vitamin B_12_ and omega-3 fatty acids could impair brain function and increase the risk for neurological and developmental disorders. The deficiency of both these nutrients can affect neural function by mechanisms [Fig. 1] discussed below.

### Homocysteine- induced oxidative stress

Elevated levels of homocysteine have been associated with vitamin B_12_ deficiency [5]. Vitamin B_12_ is an important component of the one-carbon metabolism where it acts as a cofactor for the enzyme methionine synthase, which converts homocysteine to methionine [39]. Hence, if there is a deficiency of vitamin B_12_, remethylation of homocysteine via the enzyme methionine synthase is reduced and the plasma levels of homocysteine are elevated [40]. It has also been observed that elevated homocysteine levels are a sign of disturbed remethylation of homocysteine [41].

It has been reported that homocysteine activates glutamate receptors by acting as an agonist at the glutamate binding site of the N-methyl-D-aspartate receptor [42, 43]. Thus, overactivation of the glutamate receptors leads to increased intracellular calcium levels and further activation of signaling kinases resulting in neurodegeneration and neuronal damage through a process called excitotoxicity [44, 45]. Homocysteine also increases reactive oxygen species generation and induce neuronal DNA damage, triggering apoptosis and affects synaptic and glial function [46, 47].

It is known that the brain is highly susceptible to oxidative cellular damage due to high metabolic load and poor antioxidant defense system [48]. Reports suggest that omega-3 fatty acids are susceptible for degradation due to increased oxidative stress [49]. Our earlier animal study has demonstrated increased plasma homocysteine levels in the offspring as a consequence of maternal vitamin B_12_ deficiency [50]. Further, reduced plasma levels of vitamin B_12_ and DHA and increased homocysteine levels were also observed in schizophrenic patients [51] suggesting their role in the psychological abnormality underlying the disease. We have also observed a negative association between maternal plasma homocysteine and erythrocyte DHA levels in pregnancy complications like preeclampsia [52].

Hyperhomocysteinemia through the mediation of oxidative stress produces changes in structure and function of cerebral blood vessels [53]. Animal studies have also reported that high levels of homocysteine cause damage and leakage to hippocampal microvasculature [54, 55] leading to vascular remodeling which could disrupt the blood–brain barrier [56]. Reports have also suggested that homocysteine inhibits angiogenesis through the inhibition of vascular endothelial growth factor (VEGF) and its downstream signaling pathway as demonstrated in cultured human umbilical vein endothelial cells [57]. A recent study has demonstrated beneficial effects of omega-3 fatty acid supplementation against cerebral ischemia and has been shown to enhance cerebral angiogenesis [58]. Thus, supplementation of vitamin B_12_ and omega-3 fatty acids together may help to protect against homocysteine-induced adverse neurodegenerative effects.

### Altered neurotrophins

Vitamin B12 has been implicated in the maintenance of equilibrium between neurotrophic and neurotoxic factors in the central nervous system [59]. Neurotrophins are growth factors that influence the proliferation, differentiation, survival and death of neuronal and non-neuronal cells. A series of our animal studies have demonstrated reduced levels of neurotrophins like NGF (nerve growth factor) and BDNF in the brain as a consequence of vitamin B_12_ deficiency [50, 60, 61]. The reduction in the levels of neurotrophins could be attributed to increased oxidative stress and decreased DHA levels [62].

Reduced levels of BDNF have been widely implicated in the pathophysiology of various psychiatric disorders like schizophrenia [63] Alzheimer’s [64] and Parkinson’s disease [65] and Huntington’s disease [66]. Studies have also reported lower serum NGF levels in the schizophrenic patients [67]. Lower levels of BDNF level in the schizophrenic patients has been associated with cognitive impairment [68]. It has been suggested that high neurotrophin expression in the brain may act as neuroprotective against neurological diseases [69].

Experimental evidence suggests that omega-3 fatty acids act as neuroprotective agent against neurological insults through the BDNF signaling pathways [70, 71]. It has been demonstrated that DHA supplementation in aged mice improved cognitive dysfunction through increased BDNF levels [72]. DHA is suggested to increase neurotrophins in the brain by increasing membrane fluidity, reducing oxidative stress, through neuroprotection D1 [38]. A recent study reported by us has demonstrated that combined supplementation of both vitamin B_12_ and omega-3 fatty acids together increases the levels of BDNF in the cortex and hippocampus region of the brain [18]. Thus, based on all above facts, altered neurotrophins and their downstream signaling pathway could be one of the possible mechanisms affected by the deficiency of vitamin B_12_ and omega-3 fatty acids.

### Altered angiogenic factors

**Fig. 2:**
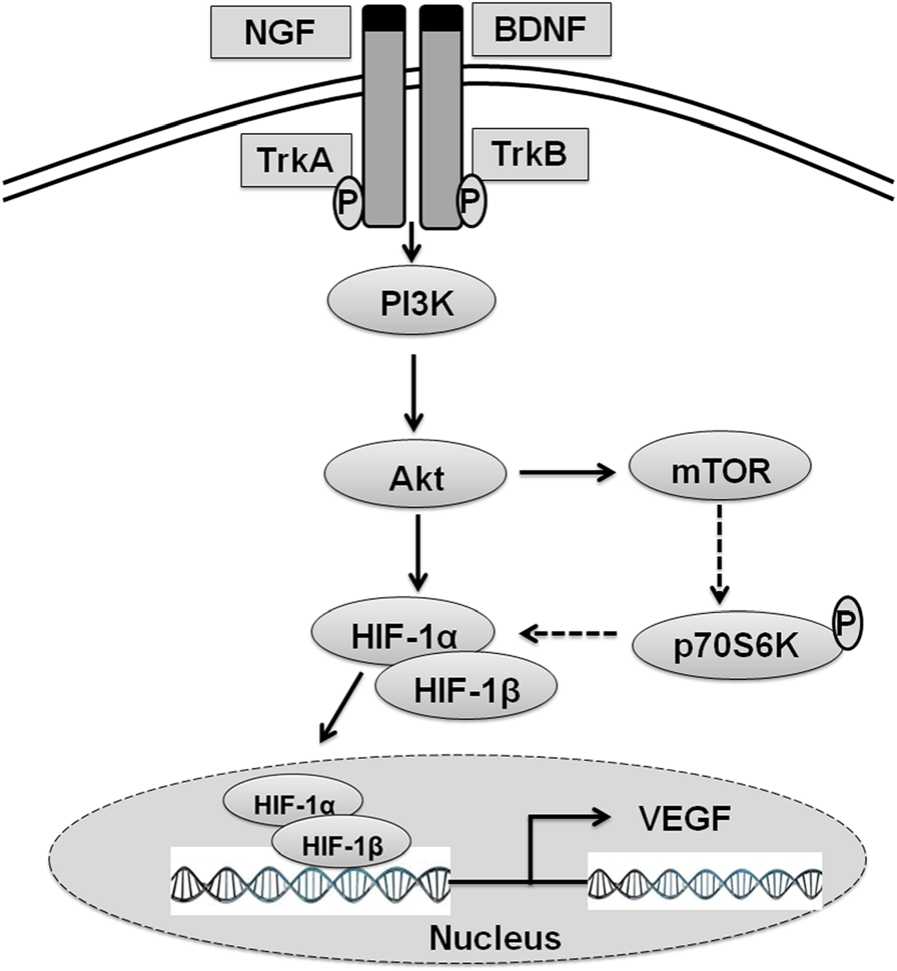
Interaction between neurotrophins and VEGF. Binding of neurotrophins like BDNF (brain derived growth factor) and NGF (nerve growth factor) to their respective receptors TrkB/A triggers PI-3kinase/AKT/mTOR pathway and leads to increased expression of HIF-1α protein expression. Activated mTOR leads to the phosphorylation and activation of ribosomal protein S6 kinase (p70S6K) which in turn known to regulate HIF-1α expression at the translational level. HIF-1α then dimerizes with HIF-1β to form HIF complex which translocate to the nucleus and binds to hypoxia response elements and leads to the increased transcription of VEGF (vascular endothelial growth factor) in the brain. NGF: Nerve Growth Factor: BDNF: Brain Derived Growth Factor; TrkB/TrkA: Tropomyosin receptor kinase B/A; PI3K: phosphoinositide 3-kinase; Akt: serine/threonine-specific protein kinase also called as protein kinase B; mTOR: mammalian target of rapamycin; HIF-1α: Hypoxia Inducible Factor-α; HIF-1β: Hypoxia Inducible Factor-β; p70S6K: ribosomal protein S6 kinase; VFGF: Vascular Endothelial Growth Factor

It has been demonstrated that neurotrophins like BDNF and NGF are involved in the regulation of angiogenic markers in the brain [73, 74]. Studies also indicate that neurotrophin activation of tyrosine kinase receptors stimulates an increase in vascular endothelial growth factor (VEGF) transcription in neuronal tissue [75]. VEGF plays a key role in promoting and coordinating angiogenesis during development and adulthood [76]. However, both in vitro and in vivo experiments indicated the diverse roles of VEGF-A in the brain including neuronal survival and migration [77]. The neurotrophin mediated increase in VEGF in neuronal cells is shown to be accompanied by an increase in the hypoxia inducible factor-1 alpha (HIF-1 alpha) levels which is dependent on tropomyosin receptor kinase (Trk)/ phosphoinositide 3-kinase (PI-3kinase)/ serine/threonine-specific protein kinase (AKT)/ mammalian target of rapamycin (mTOR) pathway [78]. HIF-1α expression is known to be regulated by the mTOR signaling pathway [79]. Activation of mTOR leads to the phosphorylation of two downstream effectors: ribosomal protein S6 kinase (p70S6K) and eukaryotic initiation factor 4E-binding protein-1 (4E-BP1) [80, 81]. Phosphorylation activates p70S6K and inactivates 4E-BP1 which in turn known to regulate HIF-1α expression at the translational level [79] (Fig. 2).

Thus, it is clear that there is an interaction between neurotrophins and VEGF in the brain. Our recent study demonstrates that maternal vitamin B_12_ and omega-3 fatty acids influence the levels and expression of VEGF and NGF in the pup brain [82].

### Altered one-carbon metabolism and epigenetic regulation

The dysregulation of the one-carbon metabolism is well implicated in brain disorders like schizophrenia, bipolar disorder, autism and depression [83]. Vitamin B_12_ is important cofactor in one carbon cycle and is involved in the formation of S-adenosyl methionine (SAM). SAM is a universal methyl donor for important methylation reactions including methylation of DNA, neurotransmitters and phospholipids. Phospholipids utilize methyl groups for the conversion of phosphatidylethanolamine (PE) to phosphatidylcholine (PC). The conversion of PE to PC in biological membranes is critical for mobilization of DHA from liver to plasma and brain [84, 85]. A study in the patients of Alzheimer disease demonstrated that the high levels of circulating homocysteine and decreased mobilization of DHA from the liver into plasma and peripheral tissues may contribute to cerebrovascular and neurodegenerative changes [86].

The one-carbon metabolism is known to influence epigenetic modifications which in turn produce long-term changes in the brain affecting memory, learning, cognition and behavior [87]. Epigenetics induces changes in the chromatin without disrupting the basic DNA sequence [88]. DNA methylation is the most widely studied form of epigenetic modification which occurs through one-carbon metabolism. DNA methylation/demethylation plays an important role in learning and memory as suppression of DNA methylation has been associated with impaired long term potentiation [89] suggesting a critical role for epigenetic modifiers in neurodevelopment [90]. Studies have demonstrated an association of memory with changes in DNA methylation in the BDNF gene [91]. It has been demonstrated that DNA methylation can also control BDNF expression during development of the forebrain in mice [92].

It is reported that adequate supply of nutrients which are the source of methyl groups to the brain is necessary for proper functioning [93]. Vitamin B_12_ is an important modifier of epigenetics being involved as a cofactor in the one-carbon cycle. It has been indicated that omega-3 fatty acids are also known to influence epigenetic mechanisms regulating gene expression [94]. We have demonstrated altered global methylation patterns in the brain of the offspring as a consequence of imbalanced (excess folate and vitamin B_12_ deficient) maternal micronutrients in animals. We also demonstrate the important role of prenatal omega-3 fatty acids in reversing methylation patterns thereby highlighting its contribution in neuroprotection and cognition [95].

Studies suggest that the brain has significantly higher levels of methylated DNA in comparison to tissues like the placenta [96]. The presence of methylated CpG dinucleotides is specific for each brain region and for each stage of development [97]. In spite of this, limited studies have examined the association of nutrients, especially those that are part of the one carbon cycle, and methylation changes in the brain of the offspring.

## Conclusion

Inadequate nutrition can increase the risk of developing neurodevelopmental and cognitive deficits. There are number of studies and reviews which have evaluated the neuroprotective benefits of vitamin B_12_ and omega-3 fatty acids in isolation, however, the combination of these nutrients are not reported. Thus, there is a need to study whether there exists any synergistic or antagonistic effects between these nutrients. Further research is recommended to investigate the optimal dose required to demonstrate preventive effects on cognitive function.

The current article discusses various mechanisms through which vitamin B_12_ and omega-3 fatty acids can support brain function. Initially, animal and cell-culture studies are recommended which will help to better understand the mechanisms involved. This may provide important insights into the etiology of various neurodevelopmental disorders.

## Abbreviations

Akt: Protein kinase B
BDNF: Brain derived neurotrophic factor
CNS: Central nervous system
DHA: Docosahexaenoic acid
HIF-1 alpha: Hypoxia inducible factor-1 alpha
mTOR: mammalian target of rapamycin
NGF: Nerve growth factor
PC: Phosphatidylcholine
PE: Phosphatidylethanolamine
PI3K: Phosphoinositide 3-kinase
SAM: S-adenosylmethionine
TrkB/TrkA: Tropomyosin receptor kinase B/A
VEGF: Vascular endothelial growth factor
